# Age-stratified clinical phenotypes of cardiac implantable electronic device infections: a 20-year retrospective cohort study

**DOI:** 10.1186/s12872-026-06736-2

**Published:** 2026-11-03

**Authors:** Sameer Al-Maisary, Migdat Mustafi, Gabriele Romano, Matthias Karck, Rawa Arif, Patricia Kraft, Mario Jesus Guzman-Ruvalcaba

**Affiliations:** 1 Department of Cardiothoracic Surgery, University Hospital of Saarland, Kirrberger Straße 57, 66421 Homburg, Germany; 2 Department of Cardiac Surgery, Heidelberg University Hospital, 69120 Heidelberg, Germany

**Keywords:** Cardiac implantable electronic devices (CIED), Pacemaker, Artificial defibrillators, Implantable prosthesis-related infections, Aging

## Abstract

**Background:**

The widespread adoption of cardiac implantable electronic devices (CIEDs) has been accompanied by a parallel increase in severe CIED infections. Traditional risk stratification frequently relies on binary age cutoffs, which can obscure how infection phenotypes evolve across a patient’s lifespan. This study evaluated how advancing age—analyzed across ordered chronological strata—correlates with anatomical infection site, device hardware type, microbiology, and in-hospital mortality in patients with CIED infections.

**Methods:**

In this retrospective cohort study of 383 patients treated for CIED infections between 2002 and 2022, patients were categorized into five sequential age brackets (< 50 to 80–90 years). Age was modeled both categorically across these strata and continuously to estimate per-decade odds ratios (ORs) for clinical presentation, hardware type, and microbiological findings.

**Results:**

The cohort was predominantly male (75.2%) with a mean age of 67.4 years. Pacemaker involvement increased significantly with age (*p* < 0.001), whereas implantable cardioverter-defibrillator use declined (*p* = 0.036). Localized generator pocket infections demonstrated a pronounced age-dependent gradient, rising from 19.4% in patients under 50 years to 73.0% in octogenarians (*p* < 0.001; OR 1.43 per decade, 95% CI: 1.21–1.69). Conversely, systemic presentations—including bacteremia and lead endocarditis—showed no age-dependent trend. In-hospital mortality remained low and stable across age groups (*p* = 0.452) but was markedly higher in patients presenting with bacteremia or sepsis compared to those without systemic involvement (16.7% vs. 1.6%, *p* < 0.001).

**Conclusions:**

Analyzing age as a continuous spectrum reveals a distinct clinical phenotype dominated by localized pocket infections and low-power devices, an insight obscured by standard binary age cutoffs. Crucially, mortality risk was driven by systemic bloodstream infection rather than patient age itself, emphasizing that clinical surveillance should prioritize infection phenotype over chronological age alone.

**Clinical trial number:**

Not applicable.

**Supplementary Information:**

The online version contains supplementary material available at https://doi.org/10.1186/s12872-026-06736-2.

## Introduction

The utilization of cardiac implantable electronic devices (CIEDs), including permanent pacemakers, implantable cardioverter-defibrillators (ICDs), and cardiac resynchronization therapy (CRT) systems, has expanded significantly over the past decades [1]. While improving outcomes in cardiac conduction disorders and heart failure, this proliferation has been accompanied by a rise in severe CIED infections, which carry substantial morbidity and mortality [2, 3].

Historically, studies evaluating CIED infections have frequently categorized age using binary thresholds, such as octogenarians versus non-elderly patients [4, 5]. However, evaluating age as a continuous or ordered spectrum better aligns with biological aging and avoids arbitrary cutoffs that may obscure non-linear or subtle trends across the lifespan [6]. Crucially, existing literature offers limited insight into how age—when analyzed sequentially across adult life—relates to the clinical phenotype of CIED infections, including the anatomical site of infection, device hardware type, and underlying microbiology [7].

CIED infections manifest across a spectrum ranging from localized pocket involvement to systemic lead-associated endocarditis and bacteremia [8, 9]. Understanding how patient age relates to these distinct anatomical presentations and microbiological profiles is essential for refining clinical assessment across diverse age groups [10].

To address these gaps, this retrospective single-center study analyzed 383 patients treated for CIED infections between 2002 and 2022. Age was evaluated as both five ordered categories and a continuous variable to determine per-decade associations. The primary objective was to investigate the association between patient age and the anatomical site of infection, device hardware type, and microbiology. The secondary objective was to evaluate the relative associations of age and systemic infection status with in-hospital mortality [1].

## Materials and methods

### Study design, setting, and population

This is a retrospective, single-center observational study conducted at Heidelberg University Hospital. The study period encompassed two decades, analyzing procedures and admissions between January 1, 2002, and December 31, 2022. The study was approved by the institutional ethics committee. Informed consent was waived due to the retrospective nature of the study and the use of anonymized data, in accordance with the European Union General Data Protection Regulation (GDPR) Recital 26. The study is reported in accordance with the Strengthening the Reporting of Observational Studies in Epidemiology (STROBE) guidelines.

Consecutive adult patients (≥ 18 years of age) who were admitted with a diagnosis of a cardiac implantable electronic device (CIED) infection were eligible for inclusion. CIED infection was defined according to the 2020 European Heart Rhythm Association (EHRA) international consensus document, requiring the presence of major criteria (e.g., positive echocardiography for lead/valve vegetations, visible generator/lead exposure, positive blood cultures for typical microorganisms) or a combination of minor criteria (local signs of pocket inflammation, unexplained fever). Patients with incomplete hospital records precluding endpoint assessment were excluded. In cases of recurrent CIED infections in the same patient, only the first (index) infectious event within the study period was included in the analysis to avoid violating the assumption of independent observations. The follow-up period was restricted to the duration of the index hospitalization.

### Clinical endpoints

The primary clinical endpoint was in-hospital mortality. Secondary endpoints included the specific anatomical presentation of the CIED infection (localized pocket infection, lead-associated endocarditis, systemic bacteremia/sepsis) and the microbiological pathogen profile.

### Clinical phenotyping and definitions

Baseline demographic and clinical data extracted from electronic medical records included body-mass index (BMI), coronary artery disease, hypertension, prior stroke, prior myocardial infarction, heart failure, diabetes mellitus, renal insufficiency, reliance on hemodialysis, and chronic obstructive pulmonary disease (COPD). Concurrent pharmacological therapies, specifically oral anticoagulation and systemic corticosteroids, were recorded. Device-related variables extracted included the total number of indwelling leads, the number of previous CIED procedures, and the primary device type (pacemaker, implantable cardioverter-defibrillator [ICD], or cardiac resynchronization therapy [CRT] device).

### Age stratification

Age was evaluated as both a continuous and a categorical variable. For categorical analyses, patients were divided into five prespecified, ordered strata based on age at the time of the index event: <50 years, 50 to 59 years, 60 to 69 years, 70 to 79 years, and 80 to 90 years. Patients under 50 years of age were pooled into a single category to maintain adequate cell counts for categorical statistical testing.

### Microbiological and temporal grouping

Microbiological data were obtained from blood cultures, pocket tissue, and extracted lead tips. Causative organisms were classified into the following predefined groups: Staphylococcus aureus, methicillin-resistant Staphylococcus aureus (MRSA), Staphylococcus epidermidis, Staphylococcus capitis, other coagulase-negative staphylococci (CoNS), Enterococcus faecalis, Enterococcus faecium, Streptococcus species, and Gram-negative bacilli. Polymicrobial infections were defined as the isolation of two or more distinct microbial species from relevant cultures; in such cases, each isolated species was recorded independently for proportional pathogen analysis.

To evaluate temporal trends in causative flora, infectious events were grouped into four prespecified epochs based on the date of the index procedure: 2002–2007, 2008–2012, 2013–2017, and 2018–2022.

### Data management and pre-processing

Data were collected and managed using a secure, dedicated electronic database separate from the statistical software. Prior to statistical analysis, data validation was performed to identify out-of-range continuous variables and transcription errors. Data completeness was audited prior to analysis; all included baseline characteristics, device parameters, and primary and secondary clinical endpoints achieved 100% data capture (0% missingness) across the 383 included patients.

### Statistical analysis

The primary statistical objective was to assess the association of patient age with CIED infection characteristics, microbiological profiles, and in-hospital mortality. All prespecified analyses were distinct from post hoc exploratory assessments.

Prior to selecting statistical tests, the distribution of continuous variables was assessed using the Shapiro-Wilk test and visual inspection of quantile-quantile (Q-Q) plots. Normally distributed continuous variables are presented as means with standard deviations, while non-normally distributed variables are summarized as medians with interquartile ranges. Categorical variables are reported as absolute frequencies and percentages.

Differences in baseline characteristics across the ordered age strata were evaluated using the Chi-square test for categorical variables; Fisher’s exact test was applied when expected cell counts were less than five. The Kruskal-Wallis test was used to compare continuous variables across age groups. To test for linear trends across the ordinal age categories, the Cochran-Armitage trend test was utilized. Monotonic associations between continuous age and continuous clinical metrics (e.g., indwelling lead burden) were evaluated using Spearman’s rank correlation coefficient (rho). Differences in microbiological composition across the temporal epochs were analyzed using the Chi-square test.

To quantify the association between advancing age (modeled as a continuous predictor) and clinical endpoints—including infection presentation phenotypes, device hardware type, comorbidity burden, and in-hospital mortality—univariable logistic regression analyses were performed. Results are presented as unadjusted odds ratios (OR) with 95% confidence intervals (CI) calculated per 10-year increase in continuous age. Unadjusted trend evaluations across ordered age strata were performed using the Cochran-Armitage trend test.

As the secondary and trend analyses were exploratory in nature, no formal adjustment for multiple comparisons was applied. A two-sided alpha level of 0.05 was predefined as the threshold for statistical significance for all tests. No formal subgroup or interaction analyses were conducted. All statistical analyses were performed using R Version 4.5.3 (R Foundation for Statistical Computing, Vienna, Austria).

## Results

### Patient cohort and baseline characteristics

The analytic cohort comprised 383 patients, ranging in age from 18 to 90 years (mean age 67.4 ± 13.6 years; median age 70 years [IQR 59–77]). The cohort was predominantly male (75.2%) and presented with a typical burden of cardiovascular comorbidities. Pacemakers were the most frequent device type (46.0%), followed by ICDs (35.0%) and CRT devices (10.4%). Pocket infection was the most common clinical presentation (38.1%), while bacteremia/sepsis was observed in 17.2% of the cohort. (Table 1).

**Table 1:** Patient demographics. Values are n (%) unless stated; continuous as mean +/- SD or median (IQR)

Characteristic	Value (*N* = 383)
Patients, n	383
Age, years - mean +/- SD	67.4 +/- 13.6
Age, years - median (IQR)	70 (59–77)
Male sex	288 (75.2%)
Body-mass index, kg/m2 - mean +/- SD	27.6 +/- 5.1
Length of stay, days - median (IQR)	8 (4–17)
Indwelling leads, n - median (IQR)	1 (0–2)
Previous CIED procedures, n - median (IQR)	2 (1–3)
Pacemaker	176 (46.0%)
ICD	134 (35.0%)
CRT	40 (10.4%)
Coronary artery disease	130 (33.9%)
Hypertension	159 (41.5%)
Prior stroke	30 (7.8%)
Prior myocardial infarction	77 (20.1%)
Heart failure	127 (33.2%)
Diabetes mellitus	93 (24.3%)
Renal insufficiency	102 (26.6%)
Dialysis	17 (4.4%)
COPD	11 (2.9%)
Anticoagulant therapy	184 (48.0%)
Systemic corticosteroids	10 (2.6%)
Endocarditis (any)	56 (14.6%)
Pocket infection	146 (38.1%)
Lead endocarditis	87 (22.7%)
Bacteremia / sepsis	66 (17.2%)
Positive blood culture	77 (20.1%)
In-hospital mortality	16 (4.2%)

Patients were stratified into five ordered age groups to examine the distribution of clinical features. The baseline distribution of the cohort was: <50 years (*n* = 36, 9.4%), 50–59 years (*n* = 65, 17.0%), 60–69 years (*n* = 86, 22.5%), 70–79 years (*n* = 120, 31.3%), and 80–90 years (*n* = 76, 19.8%)

### Infection distribution across age strata

**Fig. 1:**
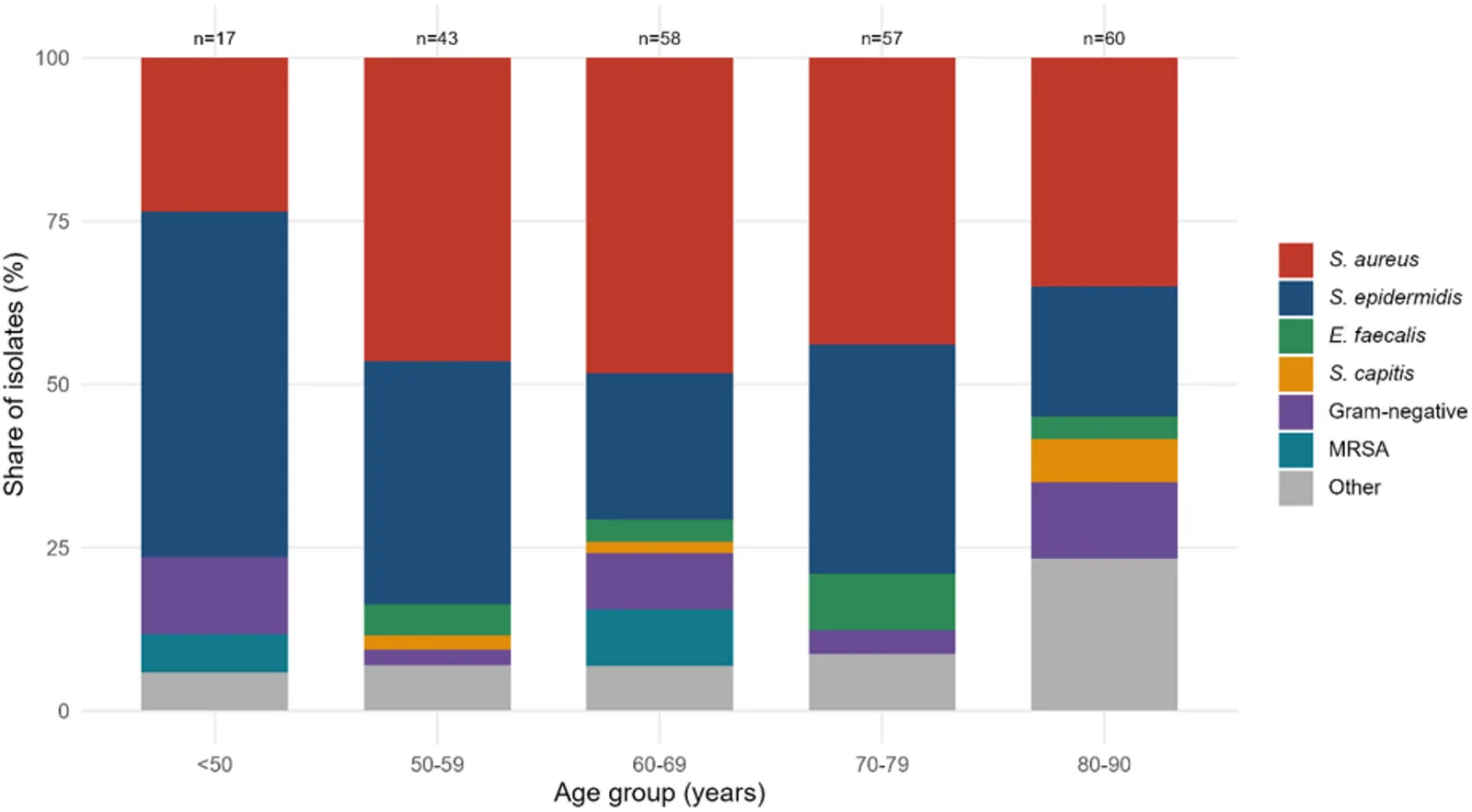
Microbiological composition by age group

Anatomical infection site varied by patient age, with localized generator pocket infections exhibiting a strong age-dependent increase from 19.4% in patients under 50 years to 73.0% in octogenarians (trend *p* < 0.001; unadjusted OR 1.43 per decade, 95% CI: 1.21–1.72; Table 2; Fig. 1). In contrast, systemic presentation phenotypes displayed no significant linear gradient across age strata, including bacteremia/sepsis (13.9% in < 50 years vs. 26.3% in 80–90 years; trend *p* = 0.085) and lead-associated endocarditis (13.9% in < 50 years vs. 28.9% in 80–90 years; trend *p* = 0.188; Table 2).

**Table 2:** Infection types and in-hospital mortality across age strata. Bold = significant linear trend

Feature	< 50	50–59	60–69	70–79	80–90	*P*-trend	OR per Decade(95% CI)
Endocarditis (any)	8/36 (22.2%)	8/65 (12.3%)	7/86 (8.1%)	20/120 (16.7%)	13/76 (17.1%)	0.791	1.09 (0.88–1.37)
**Pocket infection**	**7/36 (19.4%)**	**24/65 (36.9%)**	**28/86 (32.6%)**	**33/120 (27.5%)**	**54/76 (73.0%)**	**< 0.001**	**1.43 (1.21–1.72)**
Lead endocarditis	5/36 (13.9%)	12/65 (18.5%)	26/86 (30.2%)	22/120 (18.3%)	22/76 (28.9%)	0.188	1.16 (0.97–1.41)
Bacteremia / sepsis	5/36 (13.9%)	9/65 (13.8%)	14/86 (16.3%)	18/120 (15.0%)	20/76 (26.3%)	0.085	1.19 (0.97–1.48)
Positive blood culture	4/36 (11.1%)	15/65 (23.1%)	17/86 (19.8%)	19/120 (15.8%)	22/76 (28.9%)	0.185	1.19 (0.98–1.47)
Positive pathogen on leads	5/36 (13.9%)	9/65 (13.8%)	17/86 (19.8%)	10/120 (8.3%)	6/76 (7.9%)	0.087	0.83 (0.67–1.02)
Positive pathogen in pocket	8/36 (22.2%)	19/65 (29.2%)	24/86 (27.9%)	29/120 (24.2%)	33/76 (43.4%)	0.068	1.10 (0.93–1.30)
In-hospital mortality	2/36 (5.6%)	4/65 (6.2%)	3/86 (3.5%)	4/120 (3.3%)	3/76 (3.9%)	0.452	0.88 (0.63–1.27)

^*^P-trend calculated using the Cochran-Armitage test for linear gradients across ordered age strata. Odds ratios (OR) reflect unadjusted per-decade estimates derived from univariable logistic regression models

### Device distribution across the age spectrum

Pacemaker involvement increased linearly with advancing continuous age (47.2% in patients < 50 years to 61.8% in octogenarians; trend *p* < 0.001; unadjusted OR 1.26 per decade, 95% CI: 1.08–1.48). Conversely, ICD prevalence declined across age groups (27.8% in < 50 years to 18.4% in 80–90 years; trend *p* = 0.036; unadjusted OR 0.88 per decade, 95% CI: 0.75–1.03), while CRT device usage showed no statistically significant linear gradient (8.3% in < 50 years vs. 18.4% in 80–90 years; trend *p* = 0.141; Table 3).

**Table 3:** Device type across age strata

Feature	< 50	50–59	60–69	70–79	80–90	*P*-trend	OR per Decade(95% CI)
Pacemaker	17/36 (47.2%)	20/65 (30.8%)	27/86 (31.4%)	65/120 (54.2%)	47/76 (61.8%)	< 0.001	1.26 (1.08–1.48)
ICD	10/36 (27.8%)	29/65 (44.6%)	37/86 (43.0%)	44/120 (36.7%)	14/76 (18.4%)	0.036	0.88 (0.75–1.03)
CRT	3/36 (8.3%)	8/65 (12.3%)	4/86 (4.7%)	11/120 (9.2%)	14/76 (18.4%)	0.141	1.19 (0.93–1.58)

^*^P-trend calculated using the Cochran-Armitage test for linear gradients across ordered age strata. Odds ratios (OR) reflect unadjusted per-decade estimates derived from univariable logistic regression models

### Microbiological composition and temporal stability

The microbiological landscape of CIED infections was analyzed in relation to both patient age and calendar time. *S. aureus* remained the dominant organism overall, though it was relatively underrepresented in patients under age 50 (24% of isolates). A distinct age-related gradient was observed for *S. epidermidis*, with its share of isolates decreasing from approximately 53% in the youngest patients to roughly 20% in the oldest (trend *p* = 0.024). Figure 1; Table 4 summarize these microbiological compositions across age groups.

**Table 4:** Share of positive isolates for the leading organisms, by age stratum

Organism	< 50	50–59	60–69	70–79	80–90	*P*-trend
S. aureus	4/17	20/43	28/58	25/57	21/60	0.774
S. epidermidis	9/17	16/43	13/58	20/57	12/60	0.024
E. faecalis	0/17	2/43	2/58	5/57	2/60	0.566
S. capitis	0/17	1/43	1/58	0/57	4/60	0.140

^*^P-trend calculated using the Cochran-Armitage test for categorical variables to evaluate linear gradients across ordered age strata

**Fig. 2:**
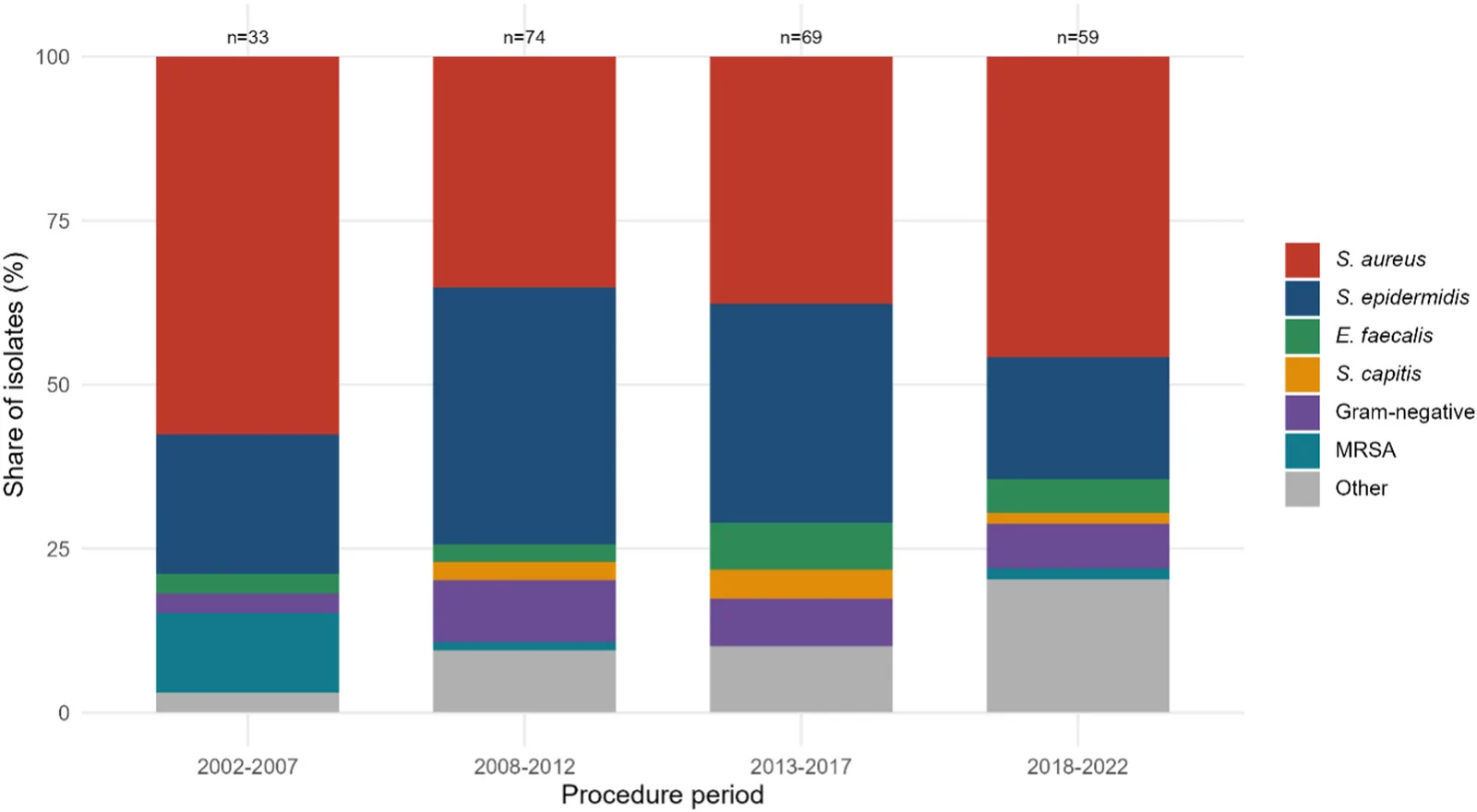
Microbiological composition by procedure period; the mix is broadly stable

**Fig. 3:**
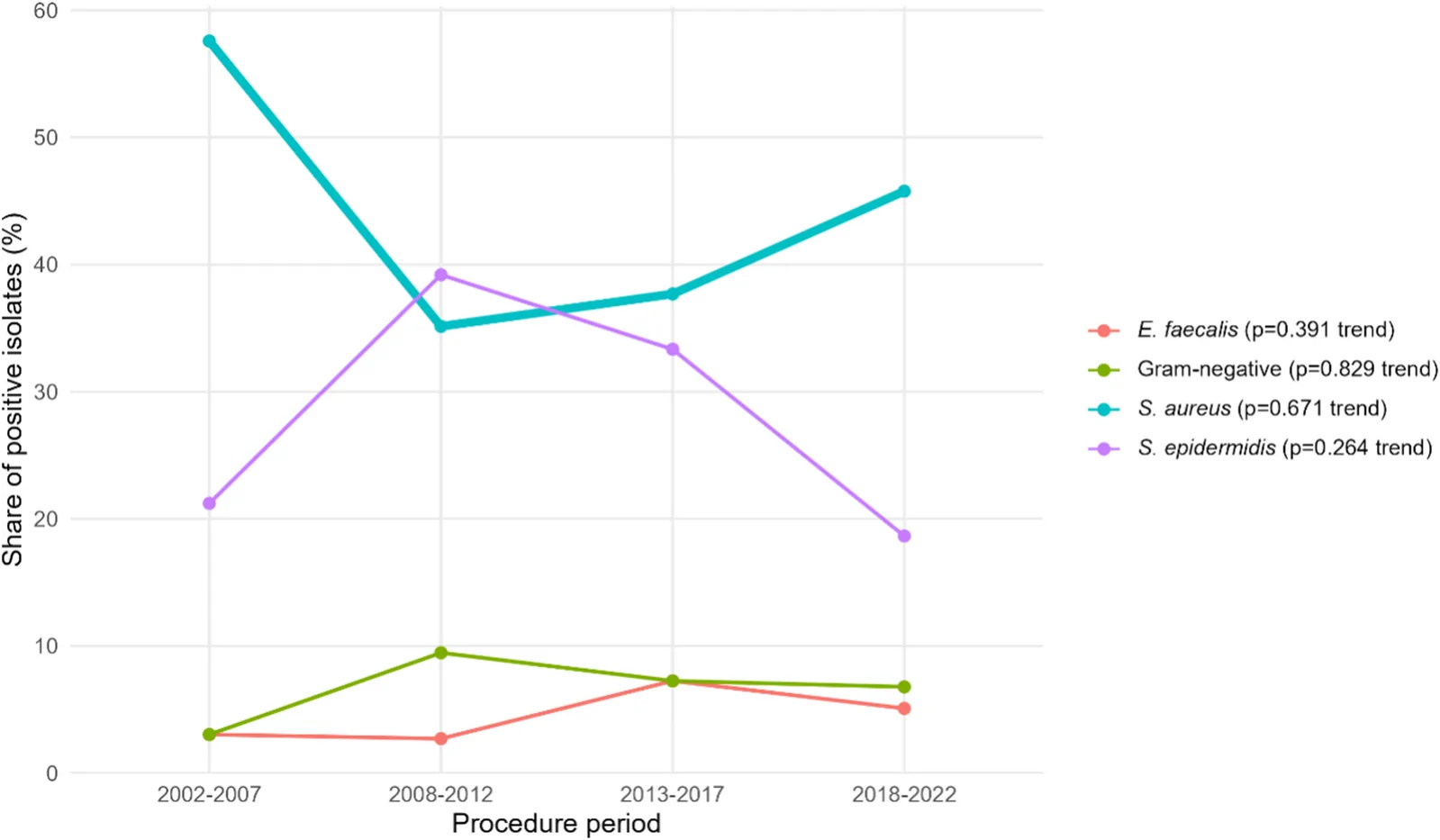
Leading-organism share by period; none shows a significant trend over time

When evaluating the stability of these organisms over the 2002–2022 period, no significant temporal trends were identified. When pooling 235 positive isolates across four time intervals, *S. aureus* consistently remained the most frequent organism (35–58%), and *S. epidermidis* remained the second most frequent; neither showed a statistically significant temporal trend (*p* = 0.671 and *p* = 0.264, respectively). The overall microbiological composition did not significantly differ across the two-decade study period (chi-square *p* = 0.067). This stability is depicted in Table 5 and in Figs. 2 and 3.

### Determinants of in-hospital mortality

**Fig. 4:**
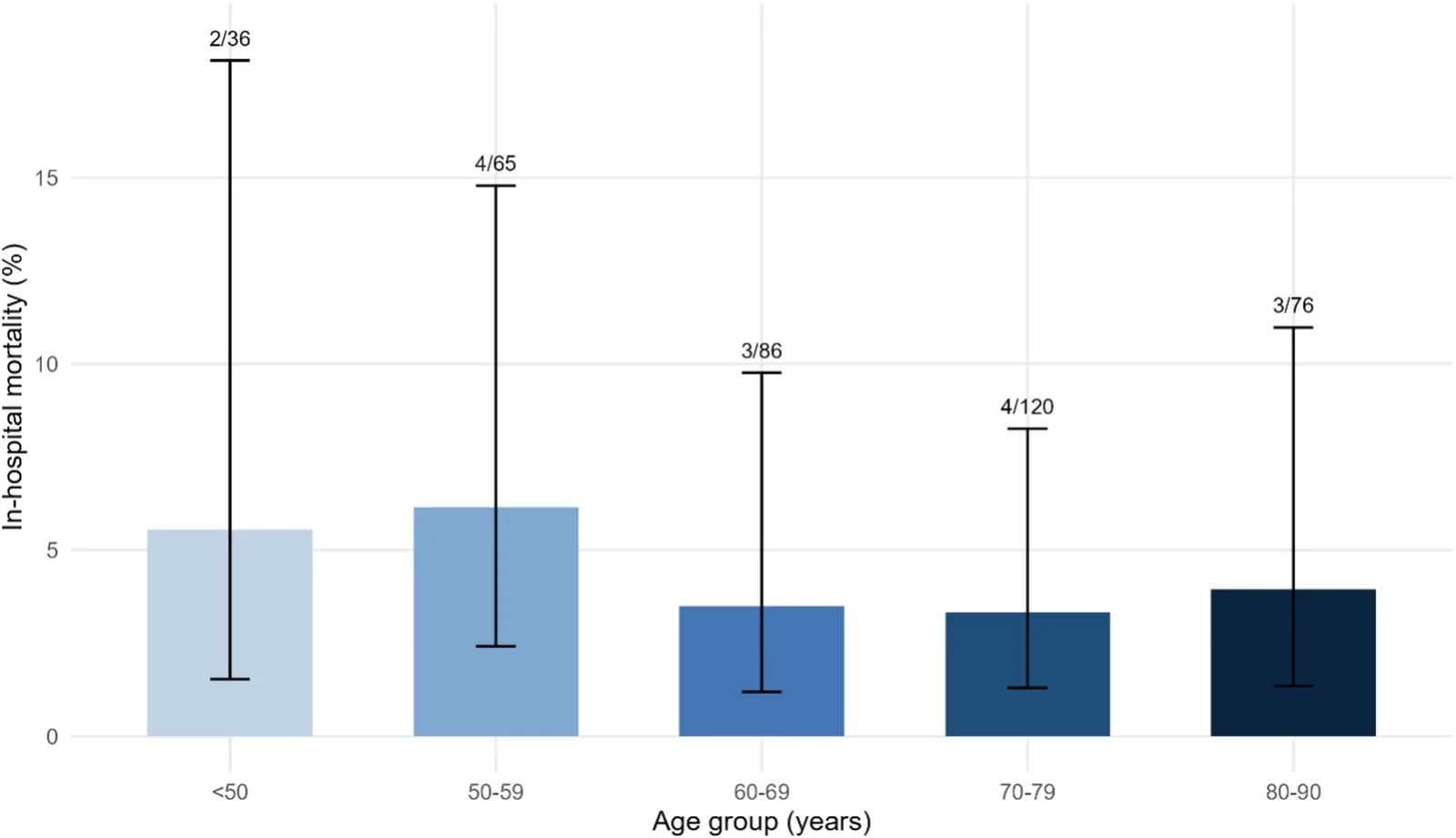
In-hospital mortality by age group with 95% CIs

In-hospital mortality remained low and stable across the entire age spectrum, with no significant trend observed (trend *p* = 0.452; OR 0.88 per decade). Figure 4 highlights this flatness in mortality rates across age groups.

**Table 5:** Organism share of positive isolates by procedure period, with the linear-trend test over time

Organism	2002–2007	2008–2012	2013–2017	2018–2022	*P*-trend
S. aureus	19/33 (58%)	26/74 (35%)	26/69 (38%)	27/59 (46%)	0.671
S. epidermidis	7/33 (21%)	29/74 (39%)	23/69 (33%)	11/59 (19%)	0.264
E. faecalis	1/33 (3%)	2/74 (3%)	5/69 (7%)	3/59 (5%)	0.391
E. faecium	0/33 (0%)	0/74 (0%)	3/69 (4%)	2/59 (3%)	0.094
S. capitis	0/33 (0%)	2/74 (3%)	3/69 (4%)	1/59 (2%)	0.660
Gram-negative	1/33 (3%)	7/74 (9%)	5/69 (7%)	4/59 (7%)	0.829
Other CoNS	1/33 (3%)	1/74 (1%)	2/69 (3%)	1/59 (2%)	0.901
Streptococcus spp.	0/33 (0%)	2/74 (3%)	1/69 (1%)	4/59 (7%)	0.092

^*^(P-trend calculated using the Cochran-Armitage test for categorical variables to evaluate linear gradients across ordered procedure periods)

**Fig. 5:**
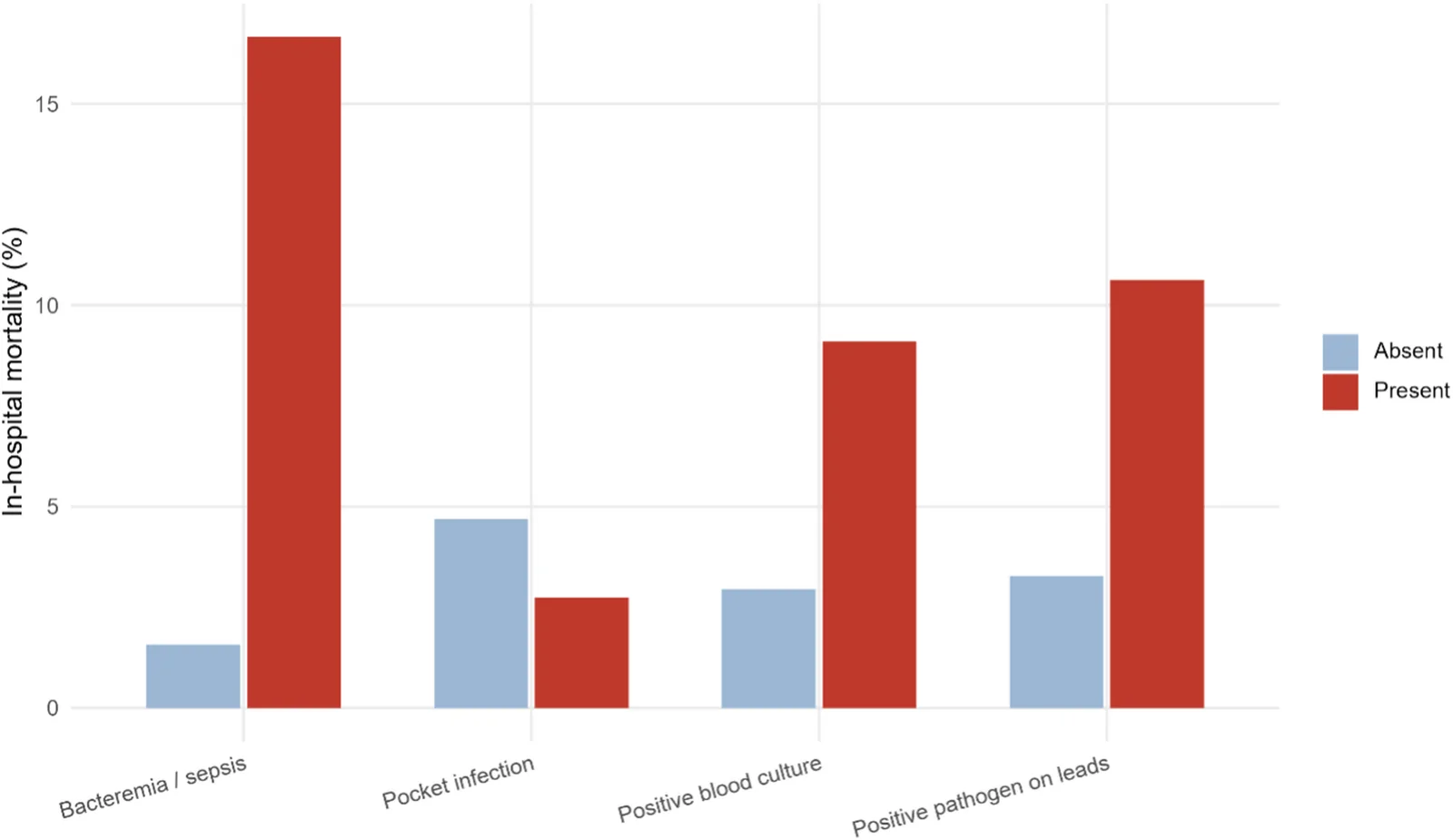
In-hospital mortality by infection feature

**Fig. 6:**
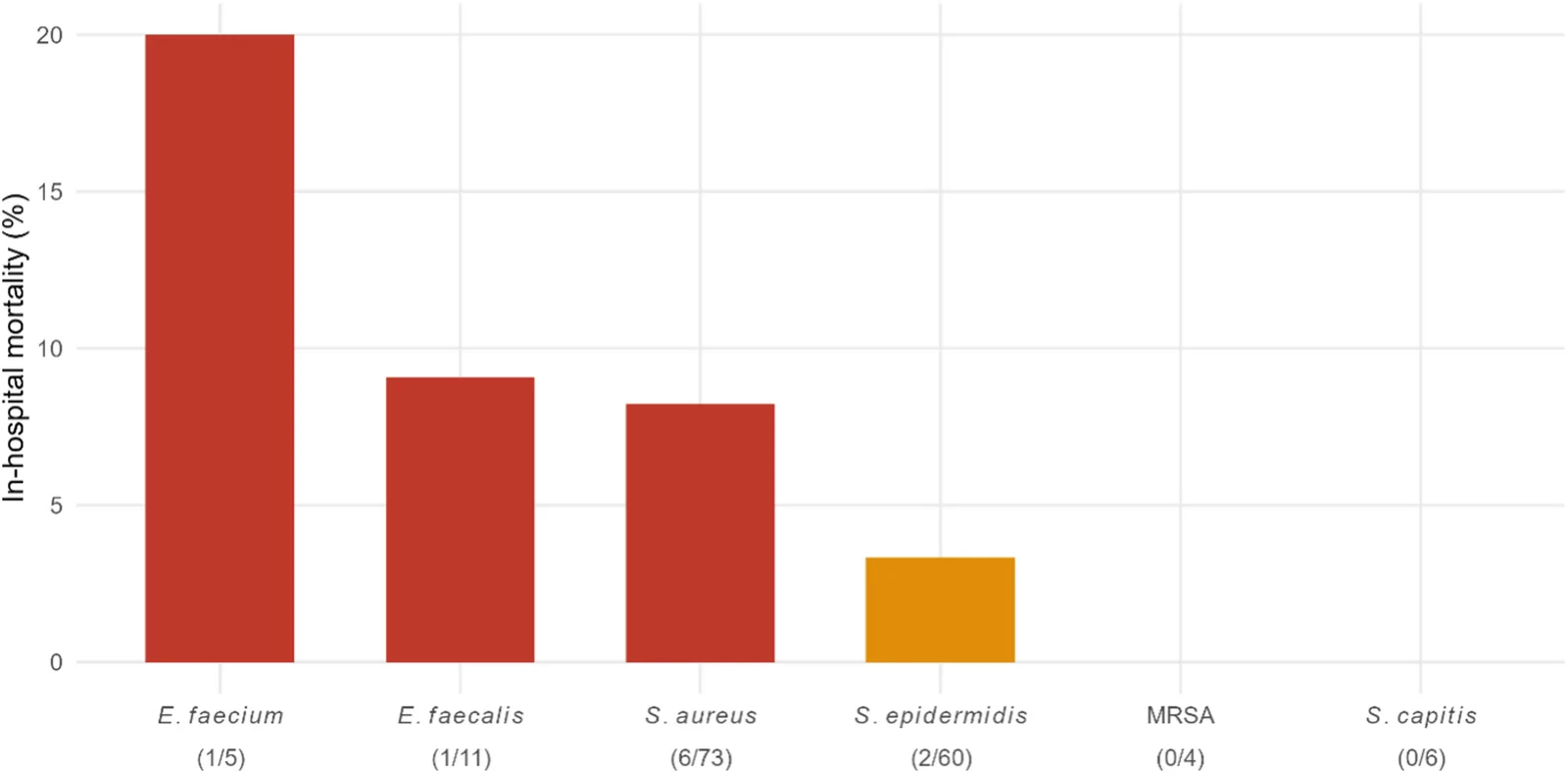
In-hospital mortality by infecting organism

In contrast, mortality was strongly linked to the type of infection, particularly bloodstream involvement. Patients with bacteremia/sepsis experienced significantly higher mortality (16.7%) compared to those without (1.6%) (*p* < 0.001). Similarly, positive blood cultures were associated with higher mortality rates. Pocket infection, despite its increasing prevalence with age, was not associated with excess in-hospital mortality (2.7% with pocket infection vs. 4.7% without; *p* = 0.425). These findings are visualized in Figs. 4, 5 and 6, which demonstrate that lethality is driven by bloodstream microbiology rather than the anatomical site of infection or patient age.

### Comorbidity and physiology gradients

**Fig. 7:**
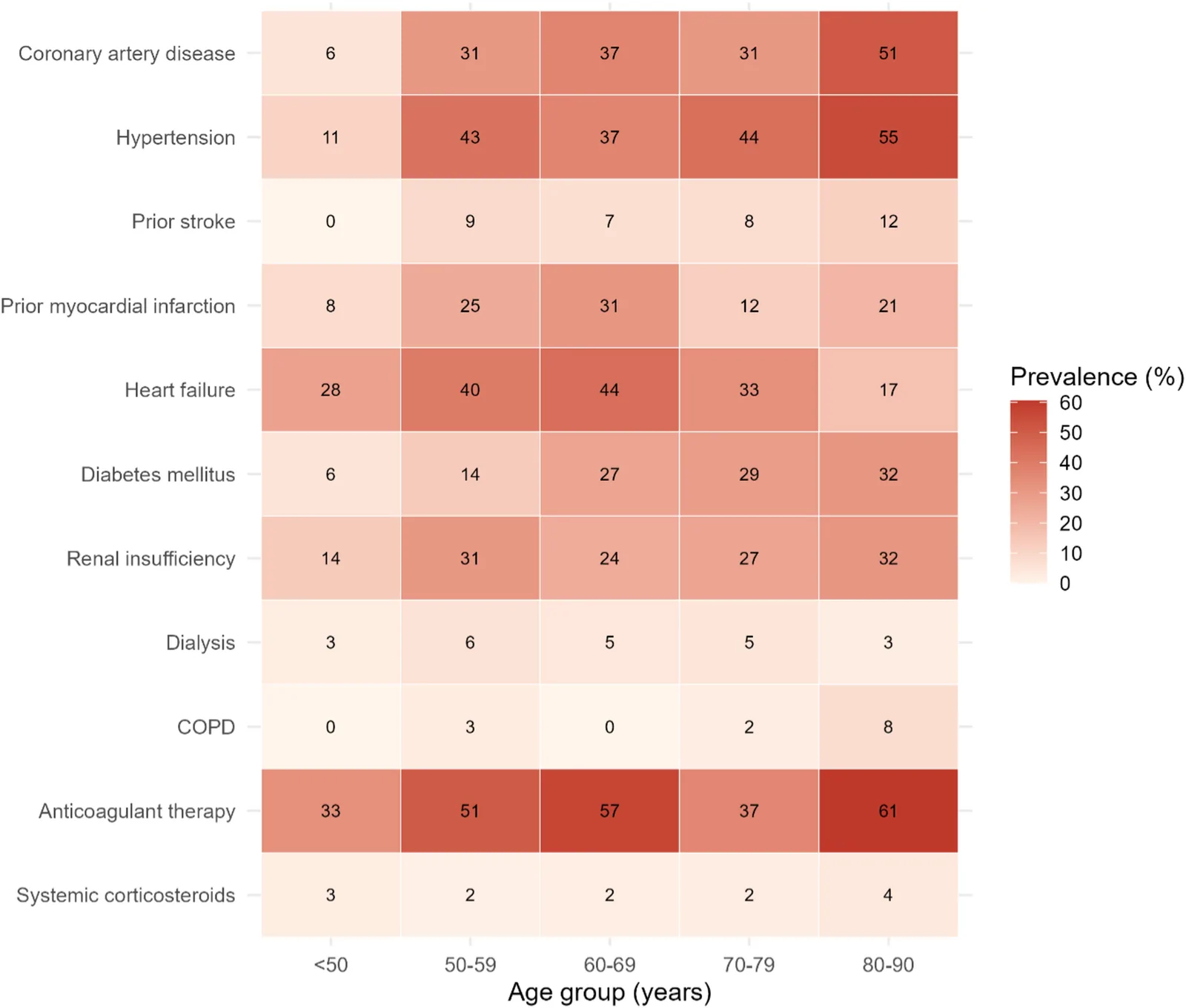
Comorbidity prevalence (%) across the age spectrum

**Fig. 8:**
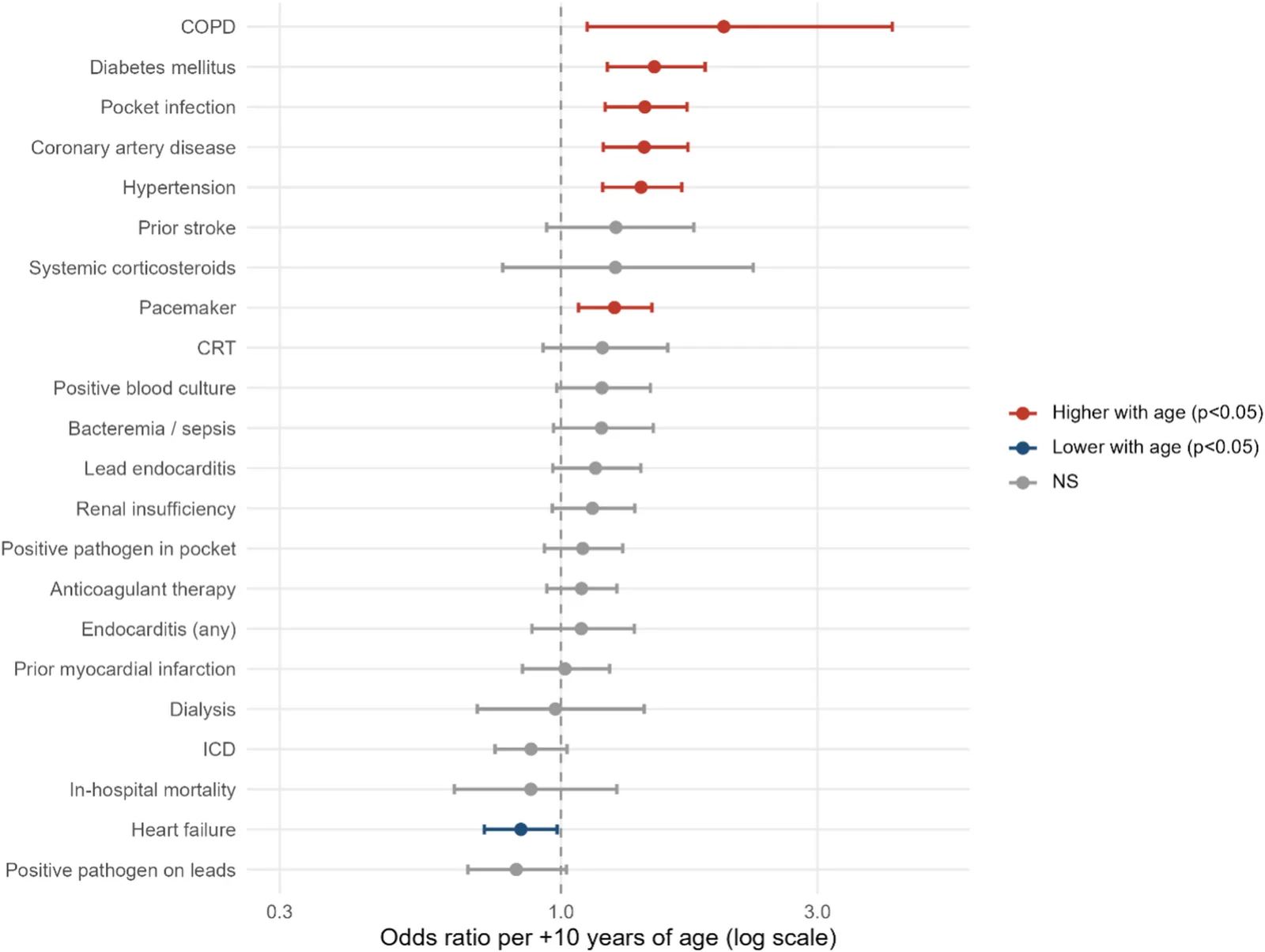
Unadjusted effect of continuous age (unadjusted OR per 10-year increase derived from univariable logistic regression models) across clinical features, device types, and baseline comorbidities

Pre-operative comorbidity burdens were examined to contextualize the findings. Coronary artery disease, hypertension, diabetes mellitus, and COPD all exhibited significant positive linear trends with increasing age (*p* < 0.001 for CAD, hypertension, and diabetes; *p* = 0.024 for COPD). Conversely, heart failure prevalence was lower in the oldest age group compared to the intermediate age groups, showing a negative association with age (OR 0.84 per decade, *p* = 0.031). These comorbidity gradients, summarized in Figs. 7 and 8; Table 6, reflect the varying clinical profiles of patients receiving different CIED therapies throughout the age spectrum.

**Table 6:** Preoperative comorbidities across age strata

Feature	< 50	50–59	60–69	70–79	80–90	*P*-trend	OR per Decade(95% CI)
**Coronary artery disease**	**2/36 (5.6%)**	**20/65 (30.8%)**	**32/86 (37.2%)**	**37/120 (30.8%)**	**39/76 (51.3%)**	**< 0.001**	**1.43 (1.20–1.72)**
**Hypertension**	**4/36 (11.1%)**	**28/65 (43.1%)**	**32/86 (37.2%)**	**53/120 (44.2%)**	**42/76 (55.3%)**	**< 0.001**	**1.41 (1.20–1.68)**
Prior stroke	0/36 (0.0%)	6/65 (9.2%)	6/86 (7.0%)	9/120 (7.5%)	9/76 (11.8%)	0.109	1.27 (0.94–1.77)
Prior myocardial infarction	3/36 (8.3%)	16/65 (24.6%)	27/86 (31.4%)	15/120 (12.5%)	16/76 (21.1%)	0.825	1.02 (0.85–1.23)
**Heart failure**	**10/36 (27.8%)**	**26/65 (40.0%)**	**38/86 (44.2%)**	**40/120 (33.3%)**	**13/76 (17.1%)**	**0.030**	**0.84 (0.72–0.98)**
**Diabetes mellitus**	**2/36 (5.6%)**	**9/65 (13.8%)**	**23/86 (26.7%)**	**35/120 (29.2%)**	**24/76 (31.6%)**	**< 0.001**	**1.49 (1.22–1.85)**
Renal insufficiency	5/36 (13.9%)	20/65 (30.8%)	21/86 (24.4%)	32/120 (26.7%)	24/76 (31.6%)	0.189	1.14 (0.96–1.37)
Dialysis	1/36 (2.8%)	4/65 (6.2%)	4/86 (4.7%)	6/120 (5.0%)	2/76 (2.6%)	0.689	0.98 (0.70–1.43)
**COPD**	**0/36 (0.0%)**	**2/65 (3.1%)**	**0/86 (0.0%)**	**3/120 (2.5%)**	**6/76 (7.9%)**	**0.024**	**2.01 (1.12–4.13)**
Anticoagulant therapy	12/36 (33.3%)	33/65 (50.8%)	49/86 (57.0%)	44/120 (36.7%)	46/76 (60.5%)	0.242	1.09 (0.94–1.27)
Systemic corticosteroids	1/36 (2.8%)	1/65 (1.5%)	2/86 (2.3%)	3/120 (2.5%)	3/76 (3.9%)	0.521	1.26 (0.78–2.28)

^*^P-trend calculated using the Cochran-Armitage test for linear gradients across ordered age strata. Odds ratios (OR) reflect unadjusted per-decade estimates derived from univariable logistic regression models

Bold = significant linear trend

## Discussion

### Reevaluating age as a continuous variable in CIED infections

Contextualizing these results against contemporary German cohort studies reveals key points of concordance and discordance that refine clinical risk prediction in CIED infections [4, 11]. In agreement with recent German surgical cohorts analyzing device management and lead extraction in older populations, our findings confirm a consistent microbiological dominance of *Staphylococcus aureus* (35–58% across epochs) and a shift in device hardware from ICDs in younger cohorts to permanent pacemakers in elderly patients [4, 11]. Furthermore, our cohort aligns with German registry data showing that complete source control via transvenous lead extraction can be performed safely across age groups, yielding low overall in-hospital mortality rates [4, 11, 12].

Conversely, important discordances emerge when comparing our continuous modeling to traditional binary studies from regional German centers [4, 11]. While previous studies comparing octogenarians directly to non-octogenarians suggested elevated procedural complexity and crude mortality in elderly cohorts [4, 5], evaluating age as a continuous spectrum demonstrates that age alone is not an independent driver of in-hospital lethality (OR 0.88 per decade, *p* = 0.452) [11]. Instead, mortality in our cohort was governed by the systemic infection phenotype (bacteremia/sepsis: 16.7% vs. 1.6%, *p* < 0.001) rather than chronological age [13, 14]. Additionally, localized generator pocket infections exhibited a progressive continuous gradient—rising linearly from 19.4% in patients under 50 to 73.0% in octogenarians—rather than a step-function risk threshold at age 80 [4, 11]. This distinction emphasizes that clinical management should be driven by bloodstream involvement and infection phenotype rather than arbitrary age thresholds [12, 15].

### Device utilization and infection phenotype across the lifespan

The distribution of device types across age groups reflects broader clinical utilization trends [16, 17]. Younger patients in our cohort more frequently presented with implantable cardioverter-defibrillator (ICD) infections, consistent with indications for primary prevention in inherited or acquired cardiomyopathies [18, 19]. Conversely, older age groups were dominated by pacemaker infections, mirroring the higher prevalence of senescent conduction disease and structural heart disease requiring pacing in later decades [11, 20, 21].

Anatomical presentation also demonstrated an age-associated shift: localized generator pocket infections accounted for a substantially larger proportion of cases in older cohorts compared to younger ones [11, 15, 21]. This observed pattern may hypothetically relate to physiological host changes—such as progressive dermal thinning, reduced subcutaneous tissue elasticity, and higher rates of anticoagulant-associated pocket hematomas—that could create a local tissue environment susceptible to erosion [17, 20–22]. However, these tissue-level mechanisms were not directly evaluated in this study and remain speculative. Also, Older patients more frequently undergo generator replacements and surgical reinterventions—established risk factors for pocket contamination [22, 23]. Differences in generator geometry and pacing lead characteristics between pacemakers and ICDs may also influence local erosion risk [11, 20]. On the other hand, younger patients presenting with ICDs or systemic involvement may experience acute lead-related complications before localized pocket erosion can manifest [18, 19].

Importantly, non-significant trends must be highlighted alongside positive findings. Intravascular lead-associated endocarditis and systemic bacteremia rates showed no linear trend with advancing age, maintaining a consistent threat level across all age strata [13, 21].

### Microbiological gradients and pathogen behavior

The microbiological spectrum remained stable over the 20-year study period, with Staphylococcus aureus functioning as the primary pathogen across all age groups [15, 16, 18, 24]. This long-term stability—despite changing prophylactic protocols, surgical techniques, and the introduction of antibacterial envelopes over two decades—underscores the persistent propensity of staphylococcal species to colonize prosthetic hardware and adhere to host matrix proteins [16, 18].

An inverse trend was observed for Staphylococcus epidermidis, which represented a smaller proportion of isolates in older patients compared to younger cohorts [11, 18, 21]. While hypotheses regarding age-related shifts in skin flora or sebaceous gland activity remain unproven in this cohort [15, 23], the primary focus should remain on clinical implications in the form of empirical antimicrobial coverage. S. aureus must remain the primary target for initial empiric therapy across all age brackets [15, 24]. Also, advanced diagnostic tools, such as plasma microbial cell-free DNA sequencing, offer promising utility for identifying pathogens in complex lead endocarditis or culture-negative systemic presentations [14, 25].

The lack of an observed association between advancing age and in-hospital mortality [11, 13] must be interpreted with caution as the overall number of in-hospital deaths was low, raising the possibility of a type II error wherein subtle mortality differences could not be detected [14]. Also, key unmeasured variables—including specific comorbidity scores, device indication, total procedure duration, and referral bias (e.g., frail elderly patients managed conservatively or referred under different criteria)—may obscure an age-related mortality effect [11, 14, 20]. The In-Hospital lethality was driven primarily by systemic involvement (septicemia or bacteremia) rather than isolated pocket infections [13, 14], highlighting that rapid source control is critical regardless of patient age [12, 15].

### Clinical implications

These observational findings support current procedural recommendations favoring complete transvenous lead extraction in the setting of systemic CIED infections. Because delayed diagnosis of systemic bacteremia is associated with mortality rates markedly higher than localized presentations, prompt recognition of bloodstream involvement remains critical. Consequently, reluctance regarding lead extraction in elderly patients based on age alone is largely unsupported. Clinical risk assessment should prioritize infection phenotype and the degree of systemic involvement to guide individualized decision-making.

## Conclusions

In this cohort, chronological age was associated with device distribution and localized infection phenotypes, but it did not independently govern in-hospital mortality. Instead, clinical outcomes were predominantly driven by infection phenotype and the extent of systemic involvement. These conclusions are limited by the study’s retrospective, single-center design. Future prospective validation is needed to confirm these age-related infection phenotypes and their prognostic impact.

## Study limitations

Several methodological limitations must be considered when interpreting these findings. First, the retrospective, single-center design carries inherent risks of unmeasured confounding, referral bias, and reliance on electronic medical documentation. Because endpoint analyses across age strata were based on univariable models and unadjusted trend evaluations, residual confounding from co-occurring clinical factors could not be fully controlled. Furthermore, these observational associations describe age-related phenotypic patterns rather than direct causal effects.Second, the relatively small sample size within the youngest age group (< 50 years) limited statistical precision and precluded a more granular assessment of younger age decades. In addition, a limited number of mortality events reduced statistical power to detect potential age-related differences in overall survival. Third, because the study period spanned two decades (2002–2022), patient outcomes may have been influenced by ongoing advancements in implantation techniques, transvenous lead extraction technologies, microbiological diagnostic methods, and infection prevention measures. Finally, conducting this study at a single center limits the generalizability of these findings to other clinical settings or non-referral patient populations. Prospective multicenter studies are needed to validate these observed age-related infection patterns.

## Supplementary information

Below is the link to the electronic supplementary material.

Supplementary Material 1

## Data Availability

All data supporting the findings of this study are available within the paper and its Supplementary Information.

## Abbreviations

BMI: Body-Mass Index
CAD: Coronary Artery Disease
CIED / CIEDs: Cardiac Implantable Electronic Device(s)
CoNS: Coagulase-Negative Staphylococci
COPD: Chronic Obstructive Pulmonary Disease
CRT: Cardiac Resynchronization Therapy
ICD / ICDs: Implantable Cardioverter-Defibrillator(s)
IQR: Interquartile Range
MRSA: Methicillin-Resistant Staphylococcus aureus
NS: Not Significant (referenced in Fig. 8)
OR: Odds Ratio
SD: Standard Deviation
